# Beyond the murmur: unmasking a rare sinus venosus ASD in an adolescent with chronic respiratory infections: A case report

**DOI:** 10.1097/MD.0000000000045437

**Published:** 2025-10-24

**Authors:** Ahmad Abdul Hakim Alhamid, Hadi Alabdullah, Mais Alreem Basel Mohaisen, Mohammad Atia, Molham Homsi, Abdulrahim Hamad Alokla, Mouhammed Sleiay, Saleh Takkem

**Affiliations:** a Faculty of Medicine, Al-Hawash Private University, Homs, Syria; b Faculty of Medicine, Hama University, Hama, Syria; c Faculty of Medicine, Al-Sham Private University, Damascus, Syria; d Department of Cardiology, National Hama Hospital, Hama, Syria.

**Keywords:** atrial septal defect, case report, inferior vena cava type, left ventricle, sinus venosus

## Abstract

**Rationale:**

Overall, 5% to 10% of all atrial septal defects (ASDs) are of the sinus venosus type, a rare congenital disease. This defect must be identified and treated immediately since it might cause significant left atrial dilatation and dyspnea.

**Patient concerns:**

A 15-year-old male presented with previously undetected sinus venosus type ASD. The patient arrived with a high-grade heart murmur and a history of recurring respiratory illnesses.

**Diagnoses:**

A clinical examination identified a 3/6 systolic ejection murmur in the second left intercostal gap and a fixed split second heart sound. Electrocardiography revealed sinus rhythm with partial right bundle branch block, while echocardiography indicated considerable enlargement of the right atrium and right ventricle, accompanied by a D-shaped left ventricle. A sinus venosus type ASD with an inferior vena cava–right atrial shunt was verified.

**Interventions:**

The patient had surgical closure of the defect via midline sternotomy, thereafter gaining access to the right atrium and performing pericardial patch repair.

**Outcomes:**

Two months postoperatively, the patient’s right ventricular diameters and pulmonary artery pressures were significantly diminished (25–30 mm Hg), and his overall status was excellent.

**Lessons:**

Sinus venosus type ASD is an infrequent etiology of persistent respiratory problems in adolescents. Early detection by thorough clinical and echocardiographic assessment, followed by timely surgical intervention, may lead to superior functional recovery and improved long-term quality of life.

## 1. Introduction

A sinus venosus type atrial septal defect (ASD) is defined by an aberrant opening in the septum between the right and left atria of the heart. This abnormality may cause the atrium’s blood flow to be disrupted, which might result in heart hypertrophy and high pulmonary arterial pressure.^[1]^ This malformation is a prevalent cardiac anomaly, occurring in around 1 in 1500 births.^[2]^ The incidence of this malformation is approximately 2 to 3 times higher in females compared to males, indicating that ASD is more commonly diagnosed in females.^[3]^ Risk factors for developing ASD include genetic predispositions and a family history of cardiac anomalies, exposure to certain environmental agents during pregnancy, such as tobacco smoke and alcohol, as well as other medical conditions. It may also arise as a result of developmental disorders during fetal growth.^[4]^ Congenital heart defects are mostly caused by genetic factors, frequently through structural changes or mutations in the genes that control cardiac morphogenesis during fetal development. Malformations may result from such genetic abnormalities interacting with the complex processes of heart formation.^[5]^ Concerning the manifestations of ASD, they may not be evident in young children and can differ based on the defect’s location and size. However, certain symptoms, including respiratory distress, an abnormal increase in respiratory rate, fatigue or weakness, elevated pulmonary blood pressure, cardiac enlargement, recurrent respiratory infections, and changes in a child’s growth rate, may be noted.^[1–5]^ In this instance, we present a 15-year-old male who presented with a history of recurrent respiratory infections and a prominent systolic murmur. Further evaluation by echocardiography revealed an inferior vena cava type sinus venosus ASD between the right and left atria.

## 2. Case report

**Figure 1.**
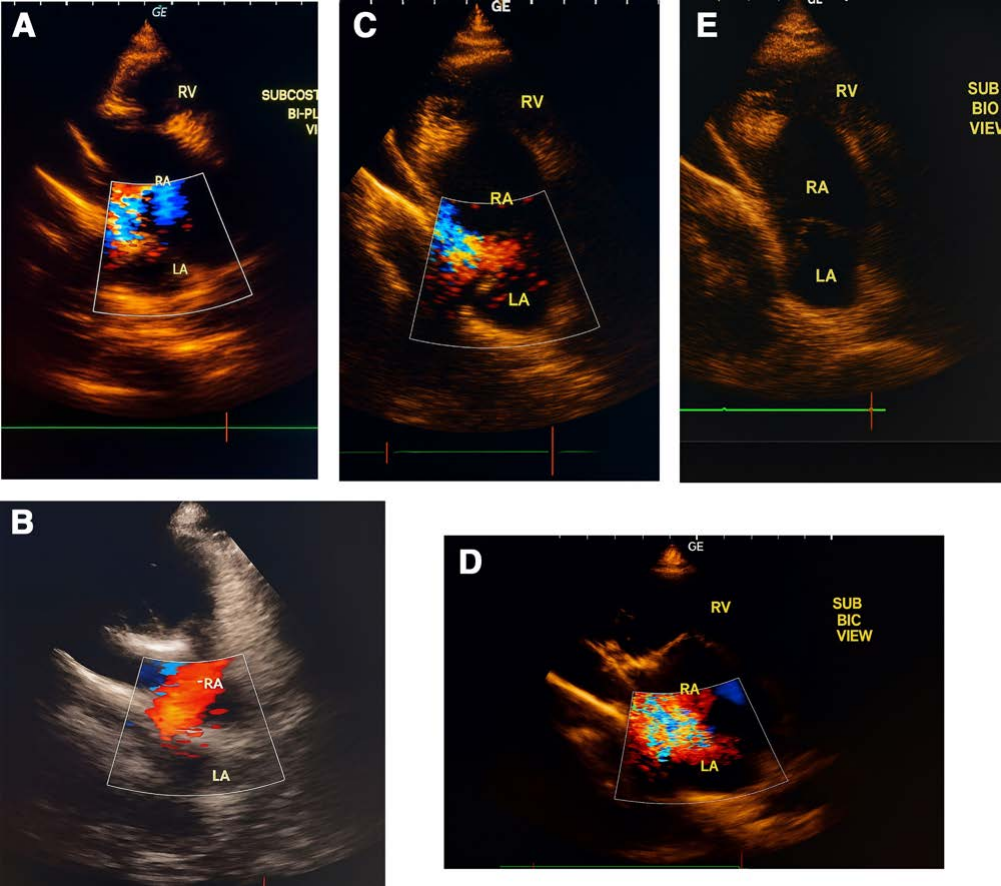
(A, B, C, D, E): This picture describes a sub-sternal view that shows the opening between the atria of the sinus venosus type near the entrance of the inferior vena cava.

**Figure 2.**
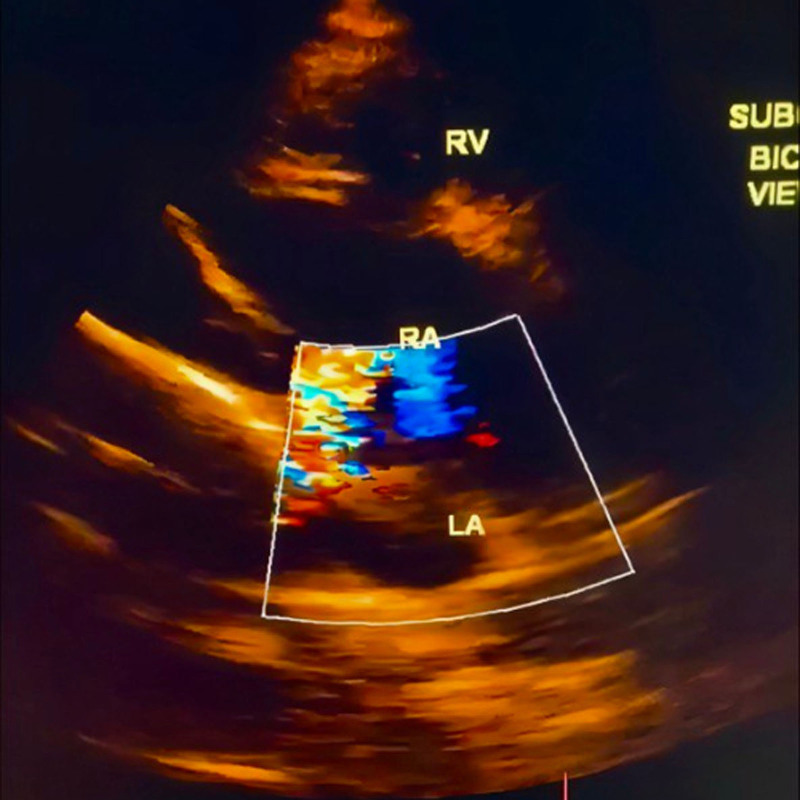
This para-sternal long axis picture in M-mode pattern shows flattening of the interventricular septum due to volume overload on the right ventricle.

**Figure 3.**
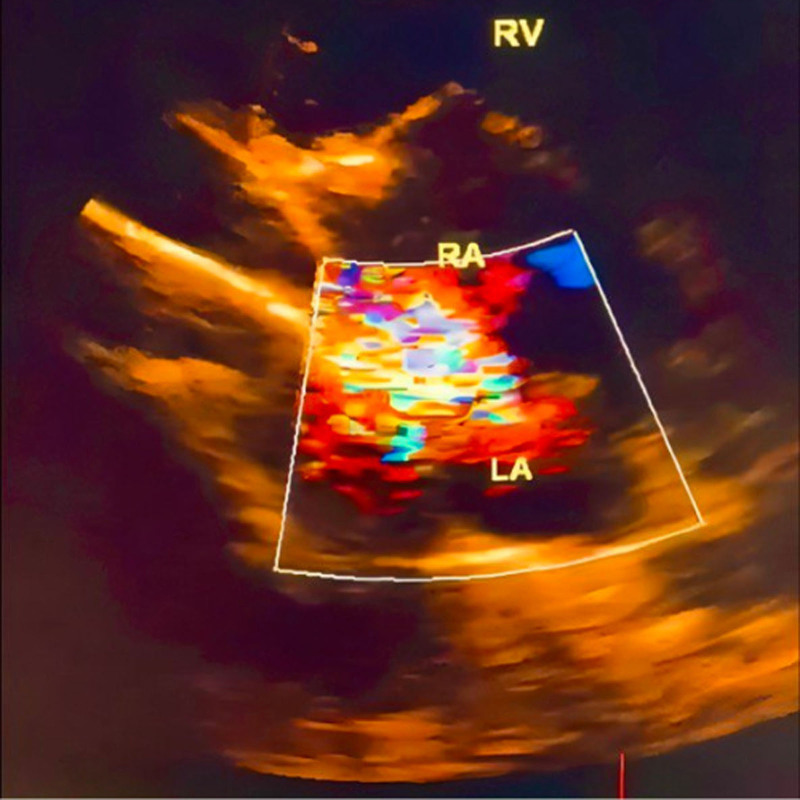
This picture describes the apical 4-chamber view that shows dilation of the right atrium and right ventricle.

A 15-year-old male arrived at the cardiology clinic with the major complaint of recurrent respiratory infections. A 3/6 systolic ejection murmur in the second left intercostal region and a fixed split second heart sound were discovered during the physical examination. The electrocardiogram revealed sinus rhythm and a partial right bundle branch block. The ejection fraction was 60%. No family history of heart disease or other illnesses exists. Moreover, the patient is a nonsmoker. The echocardiogram demonstrated moderate right atrial and right ventricular enlargement, while the left atrium remained normal (Fig. 1), and revealed a D-shaped left ventricle with preserved contractility. A sinus venous type ASD was observed (Fig. 2), in the form of an inferior vena cava-right atrial shunt (Fig. 3). The function of the right ventricle was good. There was mild systolic regurgitation (3.4 m/s) of the tricuspid valve, correlating with an estimated systolic pulmonary pressure of approximately 45 mm Hg. There was no sign of stenosis, but cardiac evaluation showed an increased flow across the pulmonary valve. The mitral and aortic valves both showed signs of regular function. Laboratory investigations indicated elevated inflammatory markers, with C-reactive protein measured at 12 mg/L and a mildly elevated serum bilirubin level of 11.7 µmol/L. Fasting blood glucose was slightly low at 72 mg/dL. Electrolyte tests, including sodium and potassium, as well as renal function tests such as blood creatinine and urinalysis, were all within normal ranges. The patient was referred for surgical closure at the cardiac surgery hospital. The surgical procedure included a midline sternotomy, followed by pericardial opening and right atrial access. Closure of the wound was accomplished using an interrupted suture with a pericardial patch. Precautions were taken to avoid compromising the inferior vena cava and pulmonary vein flow. At a 2-month follow-up, the patient’s right ventricular diameters and pulmonary pressure had decreased significantly, and their general condition was excellent. Postoperatively, systolic pulmonary pressure ranged between 25 and 30 mm Hg.

## 3. Discussion

An ASD is a congenital cardiac anomaly characterized by an abnormal connection between the right and left atria as a result of incomplete septal closure. Because this anomaly disrupts normal blood flow, oxygen-rich blood from the left atrium can combine with oxygen-poor blood from the right atrium, potentially leading to cardiovascular problems and volume overload. Based on their anatomical location and embryological origin, ASDs are divided into 3 main subtypes: sinus venosus ASD, which is responsible for nearly 10% of cases and is located closest to the junction of the superior or inferior vena cava with the right atrium; primum ASD, which is responsible for about 20% of cases and is frequently associated with atrioventricular septal defects; and secundum ASD, which is responsible for about 70% of cases and results from the incomplete fusion of the septum primum. Each type differs in clinical manifestation, hemodynamic effects, and therapeutic approaches, ranging from spontaneous resolution in minor defects to surgical or catheter-based procedures in larger, symptomatic cases.^[6]^ About 1% of congenital heart lesions are sinus venosus atrial septal abnormalities. Regarding the demographic distribution by gender and age, while no specific racial predisposition has been proven, it has been observed that females are more susceptible than males to ASDs. Less than 1 in 1000 live babies were identified with congenital heart disease in 1930; there were fewer than 0.5 live babies per 1000 that had atrial septal abnormalities found. In this report, we discuss a case involving a 15-year-old male adolescent transferred from the hospital to the cardiac clinic with complaints of recurrent respiratory infections. The fact that the patient is male makes the condition rare and distinctive. Congenital heart openings (ASDs) involving the inferior vena cava have a complex anatomical structure in comparison with the different types of congenital heart openings. One of the consequences of this deformity is the occurrence of severe thickening in the left atrium and shortness of breath, which makes it a serious disease that requires immediate intervention.^[7]^ It is important to keep in mind before beginning the diagnosis that this pathological condition causes difficulty in the closure process because the left and right atria’s connection extends to the inferior vena cava beyond the boundaries of the ventricular septum. It is possible to accurately detect heart defects, including the opening between the atria, because of cardiac echocardiography.^[8,9]^ Using transthoracic echocardiogram, we can accurately estimate the gross expansion of the inferior vena cava valve, as well as see obvious defects in the posterior atrial wall as the middle and lower pulmonary veins flow into the right atrium,^[10]^ and magnetic resonance imaging can identify the presence of an opening between the atria and accurately evaluate its size and position. Electrocardiography can also help us show schematic warning signs indicating ASD, and also by examining listening, abnormal blood flow may be associated with ASD, but it is less reliable.^[8,9]^ Therefore, in such cases, due to the young age of the affected child and the lack of various therapeutic methods, a cardiac catheterization was performed via one of the body’s large veins to access the heart and reconnect the vena cava through a covered tube from the inferior vena cava to the left atrium and from it to the right atrium through the existing hole.^[11]^ In cases that are not candidates for closure through a cardiac catheter, surgical intervention and placement of a pericardial patch or closure by suturing should be performed. The operation is performed under general anesthesia, and the child stays in the hospital for 4 to 5 days in order to stabilize his health and prevent the occurrence of plantar endocarditis.^[12]^ The patient was discharged from the hospital in good health without any complications.

## 4. Conclusions

This case report concludes by emphasizing the necessity of immediate clinical therapy and the rarity of sinus venosus type ASD, especially in male patients. Early identification with transthoracic echocardiography is critical for effective therapy, reducing right ventricular overload, and preventing permanent pulmonary hypertension and, subsequently, heart failure. Through the use of a pericardial patch for surgical correction, the right ventricular dimensions and pulmonary artery pressures were significantly reduced, improving the patient’s long-term quality of life while maintaining the flow of the pulmonary vein and inferior vena cava.

## Acknowledgments

This work was conducted under the guidance and framework of the Scientific Researchers (SRs) Organization, Damascus, Syria (e-mail: srsteamsy1@gmail.com), and we are grateful for their contribution to the coordination and resources that helped make this study possible.

## Author contributions

**Writing – original draft:** Ahmad Abdul Hakim Alhamid, Hadi Alabdullah, Mais Alreem Basel Mohaisen, Mohammad Atia, Molham Homsi, Abdulrahim Hamad Alokla, Mouhammed Sleiay, Saleh Takkem.
